# Supplementary material for: Are carbon emissions trading and green financial instruments synergistic? -Comprehensive quantitative research based on content analysis
Source: PLoS One. 2024 Mar 7;19(3):e0298601. doi: 10.1371/journal.pone.0298601 (PMC10919601; doi:10.1371/journal.pone.0298601)
Supplement: S1 Data — (ZIP) [file pone.0298601.s001.zip › Data/地级市Z_F工作报告.zip!/╣π╢1⁄2/╢1⁄2▌╕╩╨/└┤╫╘╣1⁄2╓┌║┼í╛┬φ┐╦╩2╛▌═°í┐_2006.docx]

各位代表：

　　现在，我代表市人民政府向大会作政府工作报告，请予审议，并请政协各位委员和其他列席人员提出意见。

“十五”时期的工作回顾

　过去五年，是我市综合实力强劲扩充、城市形象迅速改变、生活质素大幅提升的五年。在省委、省政府和市委的正确领导下，在市人民代表大会及其常务委员会和市政协的监督支持下，市政府始终高举邓小平理论和“三个代表”重要思想伟大旗帜，全面落实科学发展观，认真贯彻胡锦涛总书记和温家宝总理视察东莞的重要讲话精神，有效实施市委“一网两区三张牌”和“一城三创五争先”的战略思路，与时俱进，开拓创新，主动调整，努力加快发展、率先发展、协调发展，积极适应加入世贸等新形势，顺利战胜非典疫情和台风、洪涝等自然灾害，超额完成了市人大会议通过的“十五”计划任务和2005年预期目标，开创了改革开放和现代化建设的崭新局面。

　一、创新发展模式，城市实力显著增强

　 我们努力改变以往过度依赖外源型经济、以劳务出口为主、以外延扩张为主的发展模式，不断提高经济增长的质量和效益，城市实力显著增强。

　综合实力显著增强。2005年，全市生产总值2182亿元，比上年增长19.3%，五年年均增长20.2%，翻了1.4番，按常住人口计，人均生产总值3.3万元；财政总收入332亿元，比上年增长28.1%，五年年均增长26.2%，其中市一般预算收入104亿元，比上年增长25.8%，五年年均增长27.8%；金融机构本外币各项存款余额3037亿元，五年翻了1.2番。三大产业比例从2000年的3.2：54.9：41.9，调整为1.0：56.5：42.5。在近两年国家统计局的测评中，东莞连续位居“全国综合实力百强城市”的第十二位。

外源型经济实力显著增强。大力实施“外资牌”战略，优化利用外资结构，改进利用外资方式，推动招商引资向招商选资、项目招商向产业招商、圈地招商向节地招商转变。五年新签外资项目6726宗；合同和实际利用外资分别为167亿美元、133亿美元，均比前五年增长1倍。新签大项目增加，项目平均规模扩大。五年新增10家世界500强企业来莞投资，累计达到45家。2005年，全市外贸进出口总额744亿美元，五年翻了1.2番，其中出口总额409亿美元，比上年增长16.3%，五年翻了1.3番。出口商品结构优化，机电产品比重从2000年的60.3%提高到70.8%，高新技术产品比重从22%提高到36.5%。

　民营经济实力显著增强。大力实施“民营牌”战略，从放宽投资领域、培育“两自”企业等方面，扶持民营经济加快发展。五年新增私营企业和个体工商户21.4万户，累计注册户数32.2万户、注册资金535.4亿元。民营经济逐步向科技化、外向化、集团化、品牌化方向发展。全市民营企业中，省级、市级民营科技企业五年分别新增277家、870家，累计分别达到296家、1100家；取得外贸进出口经营权的企业1300家；五年新组建私营集团有限公司44家。全市现有中国驰名商标5个、省著名商标74个、中国名牌产品7个、省名牌产品69个，绝大部分属民营企业所有。

　　公有经济实力显著增强。大力推进公有经济战略性调整，对一般竞争性领域的535户市属企业，完成了以产权制度改革为中心、市属资本退出为主要形式的改革，妥善安置员工3.8万人；对关系国计民生的市属企业，改组为市属全资或控股公司制企业，促其做大做强，企业户数由2001年的11户增加到29户，资产总额由108.8亿元增加到288.6亿元；对集体经济深化改革，加强管理，2005年底全市村组两级集体总资产930.5亿元，净资产715.8亿元，可支配收入151.1亿元，分别比2000年增长82%、87.8%、64.1%。

　科技实力显著增强。大力推进科技进步，积极完善科技创新体系，加快发展高新技术产业，运用高新技术改造提升传统产业，科技实力不断增强。松山湖被评为“中国最具发展潜力高新技术产业开发区”，已引进优质企业66家，合同引资67亿美元，其中10家工业企业已经投产，广东电子工业研究院等30家研发机构和科技企业投入运作。五年来，全市的省级高新技术企业新增181家，累计247家；省级、市级工程技术研发中心分别新增9个和20个，累计分别达到12个、33个，广东生益科技建成了全市首个国家级企业技术中心；9个镇成为省专业镇技术创新试点单位。五年经国家和省市批准实施的科技计划项目1143项，获省市级科学技术奖306项，分别比“九五”增加715项、65项。2005年，全市高新技术产业产值1236亿元，专利申请6694件，分别比2000年翻了2.1番和两番。东莞连续两次被评为“全国科技兴市先进城市”。

　　二、创新发展环境，城市魅力显著增强

　我们不断优化发展环境，努力把东莞建设成为国内外最适宜创业和居住的城市之一，城市魅力显著增强，先后赢得“中国优秀旅游城市”、“国家卫生城市”、“全国绿化模范城市”、“中国最佳魅力城市”、“中国内地十大最佳商务城市”、“全国城市生活质量排行榜第二名”等荣誉。

　人居环境显著改善。大力实施“城市牌”战略，如期实现“五年见新城”目标。五年完成固定资产投资1668亿元，是前五年的4.2倍。建城方面，基本建成城市新区和松山湖科技产业园区，“三位一体”的城市框架初步拉开，依山傍水的城市格局基本成型，现代化的城市功能日益增强。基础设施网络方面，建成东江大道、东部快速、常虎高速等，新增公路352公里，新改造公路211公里，全市公路通车里程达到2871公里，公路密度达到116.5公里/百平方公里。虎门港于2003年正式对外开放，2005年进出口货物2272万吨，累计引进投资项目24个，合同投资108亿元，报建核准的已有7个深水泊位。五年全市新增日供水能力235万吨；新增主变容量2250万千伏安、输电线路1340公里、发电能力196万千瓦。2005年完成总供电量415.7亿千瓦时，五年年均增长18.5%。建设液化天然气利用工程，市区部分地区实现联网供气。五年新增固定电话用户305.8万户、移动电话用户892.7万户、互联网用户39.1万户，顺利实现固定电话号码升八位。2005年全市信息化综合指数达到71.1。整山方面，关闭采石场189家，复绿采石场65家共99万平方米；基本完成五大森林公园的建设和改造；五年市财政投入林业建设资金6.3亿元，全市林地绿化率由93.8%提高到97.2%，森林覆盖率由30.6 %提高到33.1%。治水治污方面，建成污水处理工程18项，总处理规模307.9万吨/日，2005年底全市生活污水集中处理率为13.2%，市区为72%。开征污水和生活垃圾处理费，动工兴建16座污水处理厂主体工程。建成垃圾焚烧处理工程3项，总处理规模2800吨/日，全市生活垃圾处理率44.5%，市区达到100%。整治重点污染企业，全市取缔各类违法排污企业47家，关闭砖厂206家、水泥厂47家、燃油电厂2家。市区222.5平方公里实现烟尘控制区全覆盖。全市环境质量基本保持稳定。2003年和2004年政府环保目标责任考核连续被省评为优秀。开展“四清理”，加强城市管理，提高了绿化、美化、净化、亮化水平。

　 市场环境显著改善。深化体制改革，优化市场资源配置。加快建设市场基础设施，完善商业网络，连锁、会展、物流等现代流通业蓬勃发展。全市金融机构不良贷款比例五年下降13个百分点，达到4.8%，属全省最低。完善建筑和产权等有形市场，彻底放开勘察、设计、监理等市场。全面放开网吧、沐足等特种行业经营。认真开展清费治乱减负，五年取消114项收费、降低25项收费标准，每年减轻社会负担4.4亿元。实现了全市公路基本无“三乱”。开展食品药品安全、土地市场、知识产权保护等专项整治行动，严厉打击走私贩私、骗税骗汇、违法用地、制假售假等行为，加强安全生产监督管理，全市安全生产形势基本稳定，取得整顿和规范市场经济秩序的阶段性成效。

　政务环境显著改善。结合“三个代表”重要思想、科学发展观、保持共产党员先进性等学习教育活动，建设为民务实廉洁高效的政务环境。改革政府机构，2001年市政府减少7个工作部门，精简了行政编制。改革审批制度，市级行政审批事项从1174项精简到559项。加强行政监察和审计监督，严禁政府工作人员参与“黄赌毒”，严禁收送“红包”，制止铺张浪费。建立实时在线财政预算监督系统，成立市会计核算中心和市财政投资审核中心，积极推行部门预算、国库集中收付制度等改革，规范政府采购、非税收入“收支两条线”、镇（区）财政“三集中”等制度，逐步从源头上铲除腐败滋生的条件。改革了市直单位和镇（区）机关公务用车制度。政府管理和社会服务广泛应用电子信息技术。实行市级新闻发布会和专题新闻发布会制度，进一步接受群众和媒体的监督。市政府连续两年举行“机关作风万人评”，政府机关办事效率得到提高，群众投诉有所减少。

　民主法制环境不断优化。全面推进依法行政。市政府五年制定行政规范性文件237件，提请市人大及其常委会审议通过规范性文件14件，办理市人大议案72件、市政协提案903件，做到件件有回复。年年落实市长约请人大代表、会见政协委员制度。加强执法人员上岗培训，健全行政执法责任制，规范行政执法工作。推行村（居）民自治，顺利完成了第二、三届村（居）委会民主选举。广泛开展政务公开。深入进行普法教育。调解、仲裁和法律援助工作有效加强。建立市信访督查专员等制度，落实领导包案制度，积极做好信访工作。严密防范和打击境内外敌对势力，加强流动人员和出租屋管理服务，健全社会治安防范长效机制，深入开展严打整治，五年破获刑事案件4.9万宗，查处治安案件7.8万宗，打掉犯罪团伙4255个，抓获犯罪嫌疑人5.6万人。

　　三、创新发展能力，城市活力显著增强

　我们全面实施学习型城市创建、现代市民塑造、城市亲和力强化、城市现代传媒建设、城市文化品牌打造等工程，大力创新发展能力，城市活力显著增强，先后荣获“广东省文明城市”、“全国文明城市创建工作先进城市”、“中国最具经济活力城市”等称号。

　体制活力显著增强。在全省较早建立有形土地市场、采矿权市场和土地收购储备制度，全市累计招标拍卖土地使用权132宗，成交金额81亿元；出让石场开采权17宗，收回价款1亿多元；收购储备土地1.5万亩，出库利用1780亩，回笼资金5.7亿元。深化农村体制改革，2004年在全省率先免征农业税，实现农民税费零负担；现有370个经联社和1657个经济社完成股份合作制改造，142个村委会改为社区居委会；推进农业区域化布局、专业化生产和产业化经营，培育国家、省、市级农业龙头企业14家。积极推进文化体制改革，政府管文化、社会办文化的格局初步形成。近两年实行公益文化项目招商，签约151个项目，利用民间资金1.1亿元。组建中国歌舞团东莞分团。启动文物保护机制和文艺创作繁荣机制，成功申报一批国家和省级重点文物保护单位，创作出《玉兰谣》等文艺精品，五年获得省级以上各类艺术奖项386个。改革医疗卫生体制，积极发展民办医疗机构。2005年底，全市合法医疗机构1105个，开放病床1.2万张，专业卫生人员2.2万人，分别比五年前增长22.9%、69.1%和1.4倍。

　现代文明活力显著增强。以“现代人、现代生活、现代城市”等主题教育活动为载体，积极贯彻《公民道德建设实施纲要》，大力提高公民文明素质，有效打造“开拓创新、诚信守法、团结务实、勤劳奉献”的东莞精神。优先发展教育事业，五年来市财政教育总投入42.4亿元，改善了办学条件，提高了教育质量，成功创建“广东省教育强市”。建成教育城域网和校园网，小学增设信息技术和英语教育课程。民办教育快速发展。东莞理工学院升格为本科，引进广东医学院，设立博士后工作站1个。2005年高等教育毛入学率54.5%，提前15年实现省提出的基本普及高等教育的目标。五年累计引进各类人才46.3万人，全市人才总量83.9万人，比2000年增长1.2倍。抓好人口和计划生育工作，“十五”期间年均人口出生率和自然增长率分别为10.66‰和6.11‰，低生育水平进一步稳定，人口素质有所提高。

　 就业创业活力显著增强。建立健全市、镇、村（居）三级就业服务网络，实行多项鼓励和促进就业政策，全面开展城乡统筹就业工作。2003年至2005年，累计补贴5960万元，为2.5万人提供免费或资助性技能培训，帮助4.9万人成功就业。全市城镇登记失业率控制在3%以下。从项目、融资等方面加大扶持力度，鼓励市民创业，民营经济蓬勃发展。建立东莞市留学人员创业园，入园企业40家。

　 文化活力显著增强。努力塑造开放兼容的城市精神，积极推进传统与现代、沿海与内地、东方与西洋、高雅与通俗等各种不同文化的交汇融合。投入11亿多元，建成玉兰大剧院等一批高水平的文化设施。全市现有村镇文化中心80个、图书馆（室）473个、文化广场447个、电影院和影剧院49间，初步形成市、镇、村（居）三级文化设施网络。广泛开展群众性文化活动，每年举办各类文艺晚会1000多场，各类文艺展览200多次，参与人员10万人次，观众1000万人次，打造了“文化周末”、“都市彩虹”等著名文化品牌。坚持举办“东莞学习论坛”、“东莞读书节”等活动。大力发展群众性体育运动，提高竞技体育水平。莞籍运动员五年获得国际赛金牌16枚、亚洲赛金牌8枚、全国赛金牌100枚、省赛金牌269枚。东莞荣获“全国篮球城市”称号。切实保护外来劳动者和投资者的合法权益。一百多万本地人与数百万外来劳动者、投资者和谐相处，共创繁荣。国防双拥、海洋渔业、外事侨务、民族宗教、档案方志、工青妇幼、统计、民政、气象、人防、打私、残疾等工作，也取得新的成绩。

　　四、坚持为民务实，人民生活显著改善

　我们牢固树立正确的政绩观，连续几年组织实施民心工程，全心全意为群众办实事办好事，促使人民生活持续改善，目前已经超过宽裕小康水平，正向殷实小康迈进。

　群众收入持续快速增长。2005年城市居民人均可支配收入22882元，比上年增长11.5%，五年年均增长10.1%；农民人均纯收入9842元，比上年增长8.4%，五年年均增长7.9%。城乡居民人民币储蓄存款余额1728.3亿元，五年年均增长20.8%。物价水平基本保持稳定，居民消费水平大幅提高。2005年，全市社会消费品零售总额500亿元，比上年增长17.2%，五年年均增长16.1%。城市居民恩格尔系数27.7%，五年下降4.6个百分点。城市居民每百户家庭拥有家用汽车42辆，五年增加32辆。城市居民人均住房使用面积42.7平方米，五年增加12平方米。

　社会保障体系进一步完善。积极完善职工基本养老、基本医疗、工伤、失业和生育等保险制度，推行农（居）民基本养老保险和基本医疗保险。2005年底全市各项社会保险参保者合计962.2万人次，五年增长1.4倍。完善城乡一体化的最低生活保障制度，实现应保尽保，城镇居民和农民最低生活保障标准分别提高到每人每月320元和300元。企业职工最低工资标准调高至每月574元。建立多种救助制度，积极解决困难家庭看病难、住房难、打官司难和子女读书难等问题。

　欠发达地区加快发展。五年累计发放扶贫专项资金6.4亿元，扶贫贷款23.5亿元，财政贴息1.97亿元，帮助化解债务15.7亿元，促使欠发达地区生产生活条件大大改善，近年多项经济指标的增长速度超过先发展地区。2005年，81个欠发达村净资产总额18.2亿元，比2001年增长34倍，资产负债率45.6%，下降52.4个百分点，38个村成功脱贫；11个欠发达镇生产总值合计311.6亿元，比上年增长22.3%，比全市增速快1.5个百分点。28个镇全部进入“全国综合实力千强镇”之列，并包揽第一、三、五名。五年来援助市外欠发达地区财物2.24亿元，建设希望学校104所，接收安置就业16.7万人。

　 各位代表：五年来我们取得的这些成绩，是上级党委和政府以及东莞市委正确领导的结果，是全市人民、广大外来劳动者、投资者和驻莞部队齐心实干的结果，也是与市人大、市政协的监督支持分不开的，是与香港、澳门特别行政区同胞、台湾同胞和海外侨胞以及国际友人的关心帮助分不开的。在此，我代表市政府，向所有参与、支持和关心东莞现代化建设的各界人士，表示衷心的感谢！

　五年来，我们在现代化建设中积累了许多宝贵的经验，主要有：第一，必须牢固树立和落实科学发展观。要以邓小平理论和“三个代表”重要思想为指导，确立以人为本，全面、协调、可持续的发展观，推进经济社会协调发展和人的全面发展。第二，必须始终坚持与时俱进、开拓创新的指导思想。要继续坚持打基础、办实业、走正道的优良传统，同时适应新形势，不断创新发展观念和发展思路。第三，必须加快转变经济增长方式。要以调整经济结构为重要途径，以增强自主创新能力为中心环节，以节约能源资源为有效手段，大力转变经济增长方式，努力提高经济增长的质量和效益。第四，必须加快城市化步伐。要坚持高标准规划城市，高质量建设城市，高效能管理城市，高水平经营城市，推进农村向城市、农民向市民转变，增强城市的承载力、凝聚力和辐射力，促使城市发展与经济社会发展相互协调。

　回顾过去的五年，我们也清醒地看到，全市经济社会发展中还存在不少突出矛盾和问题。主要是：粗放型经济增长方式没有根本转变，人口资源环境压力较大；自主创新能力亟待提高；产业结构不够合理，现代服务业发展相对滞后；城乡发展和区域发展仍不平衡；社会治安、安全生产形势严峻，社会管理和公共服务有待加强。这些问题，需要我们高度重视，今后重点加以解决。

“十一五”时期的奋斗目标和主要任务

　“十一五”时期，是我市建设现代制造业名城、争取率先基本实现社会主义现代化的冲刺阶段，也是我市加快推进产业升级和城市升级、全面转入科学发展轨道的关键时期。当前，经济全球化与新科技革命深入发展，推动了新一轮国际分工与产业转移。国内改革开放不断深化，产业结构和居民消费结构加快升级，区域协调发展与合作联动进一步增强，经济步入平稳快速的增长周期。我市经过改革开放20多年的建设，具备了率先基本实现社会主义现代化的基础。特别是“十五”期间的大投入、大建设，为新一轮的大提升、大突破奠定了更加坚实的基础。我们必须紧紧把握历史机遇，妥善应对各种挑战，积极开创东莞现代化建设的新局面。

　根据市委关于“十一五”规划的建议，市政府组织编制了《东莞市国民经济和社会发展第十一个五年规划纲要（草案）》，提出“十一五”时期政府工作的指导思想是：坚持以邓小平理论和“三个代表”重要思想为指导，全面贯彻落实科学发展观和构建和谐社会两大战略思想，以建设现代制造业名城、率先基本实现社会主义现代化为总目标总任务，深入实施“一城三创五争先”的战略思路，推进“四项工程”，转变发展观念，增强发展动力，提高发展质量，把经济社会发展切实转入科学发展的轨道，建设经济强市、文化新城、法治社会、和谐东莞，实现人民生活富裕安康，确保东莞现代化建设继续走在全省前列。

　“十一五”时期我市经济社会发展预期目标是：经济平稳较快发展，生产总值年均增长13%以上，力争五年翻一番；经济增长方式进一步转变，增长质量和效益明显提高，单位生产总值能耗比“十五”期末降低13%以上；经济结构更趋合理，自主创新能力显著提高，产业和城市竞争力明显增强，城乡协调发展、区域协调发展和空间整合取得重大进展；人与自然和谐相处的可持续发展环境基本建成，现代国民教育体系、科技和文化创新体系更趋完善，文化事业和文化产业更有活力，公共卫生体系更加健全；社会就业更加充分，社会保障体系更加完善，城镇登记失业率控制在3%以内；社会治安和安全生产形势进一步好转，社会更加和谐；市民普遍得到更多实惠，过上更加富足的物质生活和更加健康的精神生活，率先基本实现社会主义现代化。

　“十一五”时期的主要工作任务是：

　 一、推进产业升级，建设经济强市

　 以提高经济增长质量和效益为目标，以调整优化产业结构为主线，以提高自主创新能力为中心环节，着力推动内外源型经济做大做强与融合发展，打造经济强市。

　优化产业结构，转变经济增长方式。努力形成以高新技术产业为先导、制造业为支撑、服务业全面发展的产业格局。计划投入约311亿元，完善产业发展基础设施，引进优质产业项目，促进产业优化提升。大力发展高新技术产业，力争“十一五”期末高新技术产业增加值占工业增加值的42%，运用先进适用技术改造传统产业，推动产业高级化和适度重型化。实施“商贸东莞”工程，加强政策引导与统筹规划，提升物流、会展、旅游、批发零售和社区服务等行业的发展水平，壮大研发、会计、法律、咨询和培训等中介服务业，加快发展金融保险业，积极发展利用资本市场，稳步发展房地产业。大力发展都市型农业，投资约5.6亿元，建设10个左右农业生态园区。加快农业科技进步和结构调整，推进农业标准化建设和产业化经营。加强毛织、家具、电子信息、纺织服装、五金模具等产业集群的关联协同与技术创新，壮大特色优势与区域品牌，提升整体竞争力。加快推进松山湖、虎门港和东部工业园的开发建设，构建“三带联动、全面加速”的发展格局。

　 坚持创新发展，提高自主创新能力。计划投资约95亿元，实施“科技东莞”工程。建立健全技术创新体系，突出培育“两自”企业和建设松山湖科技产业园，侧重在电子信息技术、工业设计与集成技术、资源节约与环保应用技术三大领域，建设创新平台，大力发展高新技术产业，推进高新技术对传统产业的升级改造。加快实施制造业信息化、绿色化和标准化工程。加强国际科技合作和产学研合作。加大知识产权保护力度。加强人才培养和引进，增强自主创新能力。

　 推进内外融合，扩大对外对内开放。实行招商选资，提高引资质量，重点引进高新技术产业、现代服务业和龙头企业、地区总部以及研发中心、核心技术。实施科技兴贸、名牌带动战略，大力拓展一般贸易和出口市场多元化。优化民营经济营商环境和资本投向，鼓励其发展自主技术和自有品牌，鼓励其与外资企业合资合作经营。推动外资企业实现研发、采购、营销和管理的本土化。实施“走出去”战略。积极参与CEPA、泛珠三角和大京九协作带等区域合作。

　二、推进城市升级，建设宜居城市

　着眼于提高城市竞争力，优化空间布局，增强综合功能，进一步加快城市升级，力争到2010年，城市化水平达80%。

　 全面推进城市规划、建设和管理的升级。构筑以中心城区为主体、中心镇为支点的组团式城市框架，优化城市空间布局，加强区域统筹规划、功能互补与资源整合。建设现代化的交通、能源、供水、信息等基础设施网络，计划投资约460亿元建设综合交通工程，投资约191亿元建设能源工程，投资约50亿元建设东江与水库联网等储水供水工程，投资约110亿元建设信息化工程。构建城市管理长效机制。高标准完善城市公共设施、公共服务，积极推进平安和谐社区建设。

　加快农村城市化进程。推动交通、供水、环保等基础设施向农村延伸，教育、医疗、文化康乐等公共服务向农村覆盖。深化农村综合配套改革，稳步推进“村改居”工作，推进“城中村”的改造和农民公寓建设。认真开展农村“五整治”，即整治旧村、整治农贸市场、整治环境卫生、整治生态环境、整治“六乱”，力争一年初见成效，三年基本达标，五年全部完成。投资6.7亿元解决农村“一保五难”。继续落实扶贫政策，促进欠发达镇村加快发展。

　 加快建设资源节约型、环境友好型城市。按照废物减量化、再利用、资源化的原则，加强资源综合利用，建立循环经济政策和科技支持体系，完善再生资源回收利用体系，形成低投入、低消耗、低排放和高效率的节约型增长方式。实施“绿色制造、清洁生产”工程。严格执行土地利用总体规划和年度计划，实施土地用途管制，控制建设用地规模，推动城镇用地向中心镇集聚，工业用地向园区集聚，民宅建设向中心区集聚，零散耕地向规模经营集聚。推广环保节约型消费模式。

　 三、推进开放兼容，建设文化新城

　以提高市民综合素质为目标，积极建设文化新城，为率先基本实现现代化提供精神动力与智力支持。

　大力开展文化建设。投资约23亿元，建设教育基础设施。完善现代教育体系，高水平建设教育强市，使2007年义务教育标准化学校达标率达到100%，2010年户籍人口高中教育毛入学率达到98%以上，高等教育毛入学率达到60%以上。投资约20亿元，建设报业大厦等文化设施。投资约8.2亿元，建设一批体育场馆和体育公园。普及科学知识与文化艺术，广泛开展群众文体活动。

　深入倡导现代文明。以“爱国、守法、诚信、知礼”教育为主要内容，深入开展现代公民教育和思想道德建设，倡导现代文明生活方式与行为规范，塑造开放兼容、务实致用、开拓创新的城市人文精神。扎实推进学习型城市的创建活动，全面提升市民的现代文明素质。争取2008年创建成为“全国文明城市”。落实优抚安置政策，开展双拥共建工作，巩固军政军民团结的大好局面。

　积极创新文化体制。对国有文化企事业单位实行政事、政企分开和管办分离，优化资源配置，完善文化招商，形成多渠道、多元化的文化投入机制。加快公益性文化体育事业改革，转换机制，优化服务，推动文化、体育与经济、社会、城市和人的发展相融合。

　四、推进民主法制，建设和谐东莞

　 按照“民主法治、公平正义、诚信友爱、充满活力、安定有序、人与自然和谐相处”的要求，投资约126亿元，推进和谐社会和平安东莞建设。

　完善民主政治。积极推进社会主义民主政治的制度化、规范化、程序化。建立和完善公众参与、专家论证和政府决定相结合的行政决策机制，推进决策的民主化、科学化。健全教育、制度、监督并重的惩治和预防腐败体系，完善法律监督、民主监督和舆论监督，加强审计监督。推进村（居）民自治，完善政务、村务、厂务公开，促进基层民主建设。

　 建设法治社会。积极推进地方立规和普法工作，提高运用法律手段管理社会事务、化解社会矛盾的能力。严格依法行政，建立和完善权责明确、行为规范、监督有效、保障有力的行政执法体制。

　打造平安东莞。全面落实“打、防、管、控、建”的方针，积极推进社会治安防控体系建设。计划投入约9亿元，加强社会治安管理设施建设。建立新的大巡警体制和快速反应机制，开展多种形式的治安联防。充实基层警力，建立新的基层实战体系。实施科技强警，推进治安视频监控系统和金盾工程建设。创新流动人员和出租屋管理体制，落实管理措施。严厉打击经济领域的违法犯罪，坚决扫除“黄赌毒”。

　 维护社会公平。逐步提高最低生活保障和最低工资标准，建立困难群众医疗、教育、住房、法律等专项救助制度。加强劳动和社会保障执法，保护劳动者特别是外来工合法权益，建设稳定和谐的劳动关系。加强信访工作和矛盾纠纷排查调处，及时合理解决群众反映的问题。维护妇女儿童权益，发展残疾人事业。加强价格（收费）监管，维护正常秩序，促进公平竞争。

　 五、推进民心工程，实现富裕安康

　 坚持以人为本、执政为民，在加快发展的基础上，抓好一系列直接关系民生的重要工作。

　 积极扩大群众就业。继续实施城乡统筹的积极就业政策，进一步完善市、镇、村（居）三级公共就业服务网络、劳动信息网络平台和劳动力市场调控机制，建立健全城乡统一的就业管理制度。财政每年安排资金10亿元，主要用于促进本地劳动力就业再就业和自主创业。投资1.1亿元，支持渔港建设和渔民转产转业。

　 健全社会保障体系。以基本养老、基本医疗、失业、工伤保险为重点，以资金来源多元化、制度规范化、服务社会化为目标，建立健全涵盖全社会的社会保障体系，逐步实现农（居）民社保与职工社保统筹协调发展。构筑和健全城乡统筹的社会救助体系，鼓励发展各类慈善团体和救助基金，做好扶贫济困工作。

　 完善医疗卫生服务。投入约27亿元，加强全市医疗卫生基础设施建设。建立健全疾病预防控制体系、卫生执法监督体系、医疗救治服务体系和突发公共卫生事件应急处理机制。深化医疗卫生体制改革，进一步健全以公立医疗卫生机构为主体、民营及社会医疗卫生机构为补充的三级医疗卫生服务网络，大力推进社区卫生服务和农村卫生服务，加强药品招投标工作，规范医疗服务收费行为，逐步解决群众看病难、看病贵问题。实现“国家卫生城市”全覆盖。

　加强公共安全管理。建立健全重大疫情、重大食品安全、重大自然灾害、重大安全事故、突发事件及社会风险的预警应急机制和公共安全监控体系，构建全市应急联动指挥系统。投资约60亿元建设城市防灾减灾工程，提高防灾减灾能力。建立安全生产长效机制，有效遏制重大事故发生。投资19.8亿元，建设粮食、石油等重要物资的应急储备工程。

　 建设环保模范城市。实施“绿色制造、清洁生产”工程，建立发展循环经济的政策与科技支持体系。投资约142亿元推进生态环保工程建设，着力实施碧水、蓝天、绿地、宜居和绿色GDP五大工程，力争2007年通过国家环境保护模范城市的考核验收，“十一五”期间基本解决污水和垃圾处理问题。加强4个自然保护区和16个森林公园的建设与管理，建设生态绿城，力争城市人均绿地达到17平方米，城市建成区绿化覆盖率达到45%，陆地自然保护区占国土面积的15%。完善生态功能区划，扩大自然保护区的数量和范围。强化对自然湿地和海洋资源的管理和保护。加强生物多样性保护与生物安全管理。

2006年的工作意见

　今年是实施“十一五”规划的开局之年。政府工作的总体要求是：坚持以邓小平理论和“三个代表”重要思想为指导，坚持以科学发展观统领全局，坚持“科学发展、稳定和谐、为民务实、提高能力”的工作要求，深入实施“一城三创五争先”的战略思路，着力增强自主创新能力，推进经济结构调整，转变经济增长方式，提高经济增长质量和效益，提高城市品位，为顺利实施“十一五”规划开好局、起好步。

　今年全市经济社会发展的主要预期目标是：生产总值比上年增长15%；单位生产总值能耗下降3%左右；市一般预算收入增长15%；全社会固定资产投资总额增长25%；社会消费品零售总额增长14%；外贸出口总额增长15%；实际利用外资增长12%；居民消费价格指数上涨控制在3%以内，城镇登记失业率控制在3%以下；城镇居民人均可支配收入增长7%，农村居民人均纯收入增长6%；人口自然增长率控制在6.1‰以内。为此，必须重点抓好以下工作。

　一、加强自主创新工作，转变增长方式

　 增强自主创新能力。认真贯彻全国科技大会精神，大力推进创新型城市建设。充分用好10亿元专项资金，全面启动“科技东莞”工程，推进创新体系建设，提高自主创新能力，促进高新技术产业发展。加快建设松山湖科技产业园。以生产力促进基地为核心载体，努力引进一批国内外著名的研发机构、中试基地。加大对广东电子工业研究院的支持，加紧落实华中科技大学制造工程研究院、中国科学院东莞研究院、莞港生产力促进基地、广东工业设计院等合作项目。加强与国内外科研机构的合作，建设园区的公共信息平台、公共技术平台、检测平台、产权交易平台、认证平台和创业服务平台。推动东莞企业采用国际技术标准，鼓励企业参与行业标准、国家标准和国际标准的制定。积极开展省知识产权试点城市的相关工作，争取全年专利申请量超过7500件。积极开展国家火炬创新实验城市试点工作，共同推进科技型中小企业成长路线图计划合作协议。继续举办东莞国际科技合作周。大力引进人才团队和科研领军人物。

　扶持“两自”企业发展。认真落实《关于培育发展自有品牌自主技术企业的意见》和《东莞市企业工程技术研究开发中心管理办法》等文件，大力扶持“两自”企业发展。争取新认定省级民营科技企业超过60家，市级民营科技企业超过200家。继续实施名牌带动战略，加大品牌培育、指导、服务和保护力度。抓好大朗毛织创建国际区域品牌的试点，推动虎门服装、厚街家具等其他区域品牌的创建。

　提升制造业发展水平。用好八大支柱产业调研成果，研究制定相应的发展规划和配套政策。大力发展电子信息、电气机械、生物技术、环保和新材料等产业，争取新认定省级高新技术企业超过50家，支持35家工业龙头企业发展，催生一批集研发、生产于一体的大型高新科技企业集团，促进产业高级化。加快发展装备制造业，做大做强20家重点企业，大力发展汽车和精细化工等产业，加快建设虎门港立沙石化等大型项目，推动产业适度重型化。充分利用高新技术改造传统产业，提升传统支柱产业的竞争力，开展全市产业集群发展规划编制，抓好石龙、大朗两个省级产业集群升级示范区建设，带动全市其他产业集群发展。

　 优化三大产业结构。积极实施“商贸东莞”工程，大力发展第三产业。加大政策引导和支持力度，继续完善促进商贸流通业发展的有关措施，认真实施城市商业网点规划，优化商业布局。培育发展商贸龙头企业，运用电子信息技术改造传统服务业，大力发展物流、会展、会计、法律、信息等现代服务业，积极壮大文化、体育、旅游等产业。加快筹建东莞市保税物流中心；建设常平大京九物流基地，争取开通东莞铁路口岸至香港的货运直通列车；以虎门港开发和东江航道整治为契机，加快建立水陆联运的现代化航运体系。积极推进会展业的市场化、国际化。深化地方金融机构改革，引进外资金融机构，加大金融创新力度，努力培育并有效利用资本市场。推进“数字东莞”建设。加快发展现代都市农业。市财政安排1亿元，启动麻涌、桥头、中堂等镇农业园区的规划建设，进一步提高农业的规模化和集约化水平。设立专项扶持资金，将土地出让金纯收入的20%用于农业土地开发、农业园区和高标准基本农田建设。市财政安排500万元，力争再培育2至3家市农业龙头企业，发展1家省级农业龙头企业。

　二、加强城市建设管理，提高城市品位

　 城建重点从市区向全市延伸。一方面继续强化市区功能。启动中央生活区建设。力争8月前建成东莞理工学院体育中心，年底前建成报业大厦、市委党校新校等一批市属重点工程。动工兴建环城路北环、环莞快速路一期、东莞大道延伸段、松山湖兴园路和同沙环湖路市政连接线等。加强城市配套建设，改善给排水、交通、消防等市政设施和文体康乐设施，实施穿衣戴帽工程，改善城市功能和面貌。另一方面，将城市建设的重点扩展到全市范围。推动基础设施网络向全市延伸，市财政筹集4.5亿元用于镇区联网公路升级改造。完成龙大高速东莞段建设和北王公路改造，启动惠常高速、莞深高速三期工程，续建常虎高速新联支线，改造莞深高速黎光至东莞立交段。做好虎门港项目报批、征地拆迁等工作，加快基础设施建设。东莞东站改造升级工程列入市级重大项目，年内开工建设。做好轨道交通试验段开工的准备。投资14.7亿元，建设输变电工程51项，力争投产37项，新增主变容量494万千伏安。加快建设液化天然气工程。完成东江与水库联网储水供水工程规划设计，加快供水工程建设，力争建成市第四水厂二期管道、市第六水厂一期等工程。投资8.75亿元，完成城乡水利防灾减灾工程135宗。在中心镇优先建设重大基础设施项目，进一步增强其聚集和辐射功能。

　 基层体制从农村向城市转变。大力推进农村城市化。在全市农村实施“五整治”工程。完善城乡规划，优化生产力空间布局，有效整合土地资源，实现集约利用。大力推进镇区之间的整合与协作，对条件成熟的村组逐步实行合并。完善农民公寓建设有关配套政策，引导农民从分散居住向集中居住转变，稳步回收改造旧村庄。积极稳妥地推进“村改居”，争取上半年基本完成股份合作制改革。继续抓好“八镇百村”示范工程，提高集体经济管理水平。

　 城市管理从粗放向高效提升。加强市规划局对全市城乡规划编制的统筹职能，增强城乡规划的统筹性，提高规划水平。继续抓好全市各层次规划特别是控制性详细规划的编制和审查，建立科学完整的城市规划体系。精心组织，抓好市重点工程的规划设计。认真完成全市土地利用总体规划修编，严格控制建设用地总规模。继续深化“四清理”工作，推进城市管理强化工程。突出城市交通管理，编制全市公交总体规划，加快客货运输站场建设，完善四条主干公路配套工程和设施建设，打击非法运输，治理超限超载，保护路产路权，保证路面桥梁安全。大力加强市政管理，推进市政管理市场化。开展全市生活垃圾填埋场的评估整治工作，建立健全市、镇、村（居）、组四级市容环卫管理机构，加强市容环境卫生管理。加大价格管理和改革力度，及时查处价格违法行为。整合利用公共信息资源，力争建成工商、国税、地税、质监等部门的企业基础数据共享平台。加快实施移动信息化“八项工程”，提高城市管理智能化水平。规范行业协会管理。深入整顿市场经济秩序。

　三、加强对外对内开放，增强发展后劲

　 推动招商引资增量提质。始终把招商引资工作作为重中之重，促进外源型经济发展向规模数量与质量效益并举转变。实施产业招商，把产业关联度大、投资规模大、科技含量高的龙头项目作为主攻方向，积极引进世界500强、跨国公司和国内知名大企业、大集团到东莞设立生产中心、采购中心、研发中心和营销中心。积极落实CEPA第三阶段安排，加强莞港在物流、会展、分销以及产业支援服务业等方面的合作，争取在服务贸易领域形成新一轮的投资热潮。进一步完善重大外资项目协调推进机制，及时解决项目建设过程中遇到的问题。市统筹掌握省下达的新增建设用地年度计划指标，实行统一调剂和集中管理，重点解决首期投资在500万美元以上的项目用地。优化政府服务，促进外资企业增资扩产，支持其扩大内销业务。及时清理不合法的行政许可和收费项目，不断扩大加工贸易合同电子审批和联网监管的覆盖面，积极支持协助台心医院和台商会馆的建设，尽早完成寮步车检场的搬迁。

　推动民营经济加快发展。修订完善促进民营经济发展的政策措施，进一步加大扶持力度。继续扩大市场准入，向民间资本开放环保、交通、市政、文教体卫等领域的项目。建立民营企业与外资企业配套产品目录交流机制，引导和鼓励科技型、自主知识产权型民营企业进入外资企业的产业链。做好民营企业融资服务、上市辅导和企业家培训等工作，积极扶持民营企业50强，加快组建民营企业集团。

　 推动区域合作提升水平。用好泛珠三角、大京九等区域合作机制，继续与香港开展联合招商、联手开拓国际市场等工作，主动与广州、深圳、惠州等周边地区加强合作，协调跨境基础设施建设，共同开展污染防治等工作。用好省有关政策，加强与省内东西两翼及山区合作，稳步建设市外产业转移工业园，腾出我市空间发展高附加值产业。用好国家区域发展战略，全面加强资源、市场、人才等领域的合作，鼓励有条件的企业尤其是民营企业向中西部和东北拓展。用好广交会、东盟博览会等平台，大力开拓国际市场。积极支持和协助企业应对国际贸易摩擦。

　 四、加强人口资源环境工作，促进可持续发展

　大力发展循环经济。总结推广石龙创建科技示范镇的经验，推进“绿色制造、清洁生产”。以纺织、漂染、造纸、制革、电镀、化工、食品等行业为突破口，引导培育一批清洁生产和资源综合利用示范企业。坚持开发节约并重、节约优先，大力推进节能节水节地节材工作。加强重点用能企业管理，促进节能降耗工作。积极推广建筑节能，开发利用太阳能等可再生能源。推动企业进行环境管理体系和环境标志认证，提升工业制成品环保质量。政府机关带头开展节约资源的活动。

　加大环境保护力度。污水处理方面，力争8月底动工兴建第二批20项工程，2007年初建成首批16项工程及其截污主干管网，2007年底建成第二批20项工程。垃圾处理方面，力争8月底前动工兴建全市危险废物处理工程和长安、虎门、山区片、水乡片垃圾处理工程；年底建成全市医疗垃圾集中处理中心和全市潲水集中处理工程。启动麻涌、洪梅等9个环保专业基地的建设，迁入一批重点污染企业，实行集中建设、集中治污、集中管理。依法整治全市1252家重点污染企业，做到关闭一批、搬迁一批、管好一批；整治内河涌污染，重点是东莞运河和流经镇、村中心区河段；整治大气污染，下半年全面启动脱硫工程、机动车尾气污染整治工程以及饮食业油烟污染整治工程。

　 切实加强生态和资源保护。巩固“全国绿化模范城市”成果，创建“国际花园城市”。抓好生态环境建设重大工程。建立东莞市基本生态控制线。建设2.5万亩水源涵养林工程，营造农田林网50公里，道路绿线300公里，山上造林2.5万亩，封山育林20.5万亩，建设城市公共绿地1.5万亩，营造红树林650亩。坚持依法用地、集约用地和按规划用地，全面加强土地资源管理，严格保护耕地，盘活用好闲置土地，严格执行省委关于征地工作的“三条红线”制度，严厉打击违法用地行为。整治复绿已关闭的采石场。严禁非法采沙。加强海洋资源保护与管理。

　做好人口与计划生育工作。推进人口和计划生育综合改革，建立和完善“依法管理、村（居）民自治、优质服务、政策推动、综合改革”的管理机制，稳定低生育水平，控制人口增长速度，改善人口结构，提高人口素质。综合治理出生性别比偏高问题。加强流动人口计划生育管理和服务。落实计划生育养老保险制度。

五、加强社会综合治理，维护稳定和谐

　完善信访工作机制。全面贯彻落实《信访条例》，健全市、镇、村（居）、组（企业）四级信访工作网络，认真排查调处矛盾纠纷，探索建立解决信访问题的长效机制，切实做到调处到位、防控到位、宣传教育到位，从根本上预防和减少群体性事件的发生。

　完善治安工作机制。坚持重点防控，切实加强城乡结合部、“城中村”、出租屋和无固定职业流动人员的管理，及时消除治安隐患。坚持专群结合，广泛动员社会参与，整合治安、保安、义务巡逻队等资源，健全群防群治体系。坚持科技强警，积极推广自动报警系统和视频监控系统，年内在全市建成1700个视频监控点，筹建全市情报信息平台，逐步为所有巡逻警车安装全球卫星定位系统，全面提高110快速反应能力。年内招收1000名文职人员用于非执法岗位，从公安机关精简1000名警力下沉到基层，将现有的153个派出所合并为110个，逐步实现全市巡警人数达到民警总数的20%、基层警力达到总警力的85%、576个社区警务室都至少有1名民警专职负责。发挥公交刑警的作用，打击站场和车厢犯罪。加强各种警力协同作战，坚持“严打”整治，从重从快打击“两抢一盗”、黑恶势力以及严重暴力犯罪等。

　完善就业再就业机制。大力实施“创业东莞”工程，市政府安排10亿元，用于推荐就业补贴、企业岗位培训补贴、技能人才奖励、村（社区）促进就业奖励等。加快建立促进就业创业工作机制，完善积极就业政策体系和配套措施。继续抓好劳动普法执法工作，提高企业法制观念。启用全市工资监控、监察信息系统，加快建立企业守法诚信档案制度，推行重大劳动违法行为社会公示制度，抓好源头防范整治，大力查处拖欠工资等违法行为。进一步落实劳动合同制度，督促企业严格执行最低工资保障制度，建立健全劳动力市场工资指导价位制度，完善企业工资分配管理。将劳资纠纷信访情况列入镇村年度工作量化考核评比。

　完善社会保障机制。提高农（居）民基本养老保险基础养老金标准，7月起启动农（居）民基本养老保险与职工基本养老保险统筹发展。继续完善职工基本养老、基本医疗、工伤、失业、生育等保险制度，督促全市各类企业依法为劳动者落实参保缴费，适度扩面征缴。健全城乡统筹的社会救助体系，加大对社会救助的财政投入，切实解决农村“一保五难”问题。积极发展残疾人事业。

　 完善安全生产监管机制。建立健全各项突发公共事件应急预案，提高政府处置突发公共事件、保障公共安全的能力。建设覆盖全市的气象监测警报网络。立足防范，把亿元生产总值死亡率、工矿商贸从业人员十万人死亡率、道路交通万车死亡率等指标纳入经济社会发展考核指标体系。落实安全生产责任制，强化机构、信息和制度建设，健全安全生产网络，加大安全生产公共投入。加大执法力度，大力整治安全隐患，重点抓好学校接送车、采石场、危险化学品、水陆交通、建筑施工、食品药品、人员密集区、出租屋与“三小”场所等专项安全整治。

　 六、加强文化新城建设，促进全面发展

大力弘扬现代文明。认真学习贯彻胡锦涛总书记有关讲话精神，引导干部群众树立“八荣八耻”的社会主义荣辱观。继续深化“爱国、守法、诚信、知礼”的现代公民教育，着力开展“文明东莞三有序”活动，促进全国文明城市创建工作。开展东莞城市精神大讨论。加强和改进未成年人思想道德建设。培养科学文明的现代生活方式，推进农民市民化。努力营造和睦相助、友爱向善、谅解宽容的人文环境，增强城市亲和力，促进多元文化的交融和包容。

　推进教育现代化。加大对欠发达镇教育的扶持力度，争取年内新增省教育强镇4个。建立镇（区）教师工资保障机制。全面实行6周岁入读小学。今年秋季新学年开始，全市城乡户籍的义务教育阶段学生全部免收杂费。积极推进联合办学，调整中小学和职业学校的布局，进一步优化教育资源配置。开展全市高中招生考试改革和莞城区小学升初中均衡招生改革。加快示范性普通高中建设，争取6所学校早日成为“广东省示范性普通高中”。大力发展高等教育。推进松山湖虚拟大学园建设。抓好职业教育的调整、充实和提高。挖掘公办学校潜力，扶持和规范民办学校发展，积极解决非户籍常住人口子女入学问题。坚持人才培育和人才引进并举，打造人才强市。

　繁荣文化事业和产业。全面推进图书馆之城、博物馆之城、广场文化之城建设，继续举办东莞读书节、东莞学习论坛等活动，组织实施共享文化阳光工程。完善并经营好市、镇、村（社区）三级文化设施网络，抓好可园传统文化园区筹建工作。扶持重点文学艺术创作项目，鼓励社会办文艺团队，繁荣文艺创作。提高公益文化项目招商效益，规范基层文化建设考评。大力发展企业文化、乡村文化和节日文化，丰富群众文化生活。进一步放开文化产业经营领域，努力提高文化产业规模化、集约化和专业化水平。积极稳妥推进市属文化单位改革，强化对文化市场的监管，推进文化事业与产业协调发展。积极繁荣发展哲学社会科学事业。

　发展卫生体育等各项事业。加大投入，全面推进公共卫生“一个机制三大体系”建设，扎实做好艾滋病、高致病性禽流感等传染病和职业病的防治以及食品卫生的监管工作。坚决打击非法行医行为，保障群众的就医安全。深化医疗卫生体制改革，大力发展社区卫生服务和农村卫生服务。抓好群众体育健身设施建设，引导群众参与科学健身活动。努力提高竞技体育水平，争取在今年的省运会上取得好成绩。办好广东省第七届大学生运动会。全面贯彻新修订的妇女权益保障法，切实保障妇女和未成年人的合法权益。继续做好国防动员、民兵预备役和双拥工作，争取实现“全国双拥模范城”六连冠。认真做好民族、宗教、外事、侨务、台务、统计、气象、人防、打私、档案、方志等工作。

　 加强政府自身建设。继续推进科学行政、民主行政、依法行政和廉洁行政。大力改进机关作风，加强行政监察，建设电子政务，提高行政效能。加快事业单位机构改革。完善镇村财务管理。深入推进廉政建设和反腐败斗争，集中开展治理商业贿赂专项工作，坚决纠正损害群众利益的不正之风。发扬艰苦奋斗的作风，严禁奢侈享乐和铺张浪费。年内为群众办成十件实事：①明显改善社会治安状况；②全面解决农村“一保五难”问题；③促进城乡基础教育均衡化发展；④建成一批基础设施；⑤保护环境和生态；⑥促进就业和创业；⑦完善社会保障机制；⑧加强农产品质量安全检测；⑨全面启动水利防灾减灾工程建设；⑩保护外来劳动者的合法权益。

　各位代表，“十五”的辉煌使我们信心倍增，“十一五”的伟业更使我们豪情满怀。让我们高举邓小平理论和“三个代表”重要思想伟大旗帜，全面贯彻落实科学发展观，把握机遇，迎接挑战，团结拼搏，为全面完成今年和“十一五”的各项工作任务而努力奋斗！
